# Gold Nanomaterial System That Enables Dual Photothermal and Chemotherapy for Breast Cancer

**DOI:** 10.3390/pharmaceutics15092198

**Published:** 2023-08-25

**Authors:** Lijun Wang, Binita Shrestha, Eric M. Brey, Liang Tang

**Affiliations:** 1 Department of Biomedical Engineering & Chemical Engineering, University of Texas at San Antonio, San Antonio, TX 78249, USA; lijun.wang@utsa.edu; 2 Department of Biomedical Engineering, University of Texas at Austin, Austin, TX 78705, USA

**Keywords:** gold nanorods, gold nanoclusters, combinational therapy, photothermal therapy, chemotherapy

## Abstract

This study involves the fabrication and characterization of a multifunctional therapeutic nanocomposite system, as well as an assessment of its in vitro efficacy for breast cancer treatment. The nanocomposite system combines gold nanorods (GNRs) and gold nanoclusters (GNCs) to enable a combination of photothermal therapy and doxorubicin-based chemotherapy. GNRs of various sizes but exhibiting similar absorbance spectra were synthesized and screened for photothermal efficiency. GNRs exhibiting the highest photothermal efficiency were selected for further experiments. GNCs were synthesized in bovine serum albumin (BSA) and integrated into citrate-capped GNRs using layer-by-layer assembly. Glutaraldehyde crosslinking with the lysine residues in BSA was employed to immobilize the GNCs onto the GNRs, forming a stable “soft gel-like” structure. This structure provided binding sites for doxorubicin through electrostatic interactions and enhanced the overall structural stability of the nanocomposite. Additionally, the presence of GNCs allowed the nanocomposite system to emit robust fluorescence in the range of ~520 nm to 700 nm for self-detection. Hyaluronic acid was functionalized on the exterior surface of the nanocomposite as a targeting moiety for CD44 to improve the cellular internalization and specificity for breast cancer cells. The developed nanocomposite system demonstrated good stability in vitro and exhibited a pH- and near-infrared-responsive drug release behavior. In vitro studies showed the efficient internalization of the nanocomposite system and reduced cellular viability following NIR irradiation in MDA-MB-231 breast cancer cells. Together, these results highlight the potential of this nanocomposite system for targeted breast cancer therapy.

## 1. Introduction

Breast cancer is one of the most prevalent cancers and the second leading cause of death in females [1,2]. While chemotherapy and hormone therapy have considerably reduced the recurrence and mortality, acquired resistance and systematic toxicities remain major challenges. These major challenges can be attributed to high systemic dosage of the therapeutic agents required to achieve therapeutic dose at the diseased site and its non-specific delivery to normal cells [3]. Various types of nanoparticles including metallic, inorganic and organic nanoparticles have been explored for the loading and delivering of therapeutics at the disease site for enhanced therapeutic effects while minimizing dose-associated toxicities and multi-drug resistance [4,5,6]. Nanocomposites are engineered systems, developed by functionalizing varieties of nanoparticles with biocompatible polymers and desired therapeutic molecules to produce a stimuli-responsive and multifunctional system for anti-cancer therapy. For example, Salem et al. developed nanocomposites utilizing 5-Fluorouracil encapsulated chitosan nanoparticles loaded with gold nanoparticles for chemo-photothermal therapy for hepatocellular carcinoma [7]. Pre-clinical and clinical studies have highlighted the limited anti-cancer effect of monotherapy and emphasized the importance of combinational therapy [8]. The combinational therapy targets the cancer cells through multiple key pathways in a synergistic or additive manner while minimizing drug resistance and systemic toxicities [8,9]. Furthermore, smart nanocomposites for combinational therapy provide added spatial and temporal control over the drug release at the diseased site [10]. Of the various combinational therapies, chemo-photothermal therapy results in lower systemic side effects and reduces drug resistance [11].

Photothermal therapy (PTT) uses nanoparticles to convert absorbed light to heat to kill cells. Nanoparticles, such as magnetic nanoparticles, gold nanoparticles, graphene oxide, and carbon nanotubes, among others, are commonly used for PTT. However, their long synthesis procedure, broad size distributions, low yield, low biocompatibility, and hydrophobicity are some major limitations for magnetic nanoparticles [7], graphene oxide [12], and carbon nanotubes [13]. Gold-based nanomaterials are promising candidates for safe, convenient, and efficient PTT primarily due to their (i) biocompatibility, (ii) unique optical properties, (iii) tunable size and morphology, (iv) ease of surface functionalization, and (v) the fact that they can be used as a fluorescence probe for diagnostic purposes [10,14,15,16,17]. Gold nanorods (GNRs), among other morphologies, have demonstrated enhanced PTT efficiency. The unique optical properties of GNRs are associated with their anisotropy and rod-like shape, and controlled synthesis methods allow for tuning localized surface plasmon resonance (SPR). Due to their anisotropic geometry, GNRs exhibit transverse surface plasmon resonance (TSPR) along the cross-section dimension and longitudinal surface plasmon resonance (LSPR) along the longitudinal dimension [18,19]. The LSPR range of GNRs can be tuned between visible and near-infrared (NIR) regions from 600 to 1400 nm [15,20]. These characteristics enable GNRs to efficiently absorb and convert radiant optical energy into thermal energy [16,18], making them effective photothermal agents in various applications [17,21].

Gold nanoclusters (GNCs), a sub-class of gold nanoparticles, are also widely used in PTT [22,23,24,25]. Unlike nanorods, GNCs consist of a few to hundreds of gold atoms with discrete electronic structures. The ultra-small size and well-defined molecular structure lead to molecule-like properties, such as enzyme-like activities [26,27], fluorescence [22,28], high renal clearance, and long blood circulation [29]. These unique properties, attributed to the quantum confinement effect [30,31,32], make GNCs promising photosensitizers for hyperthermia-based therapy. In previous studies, GNCs were reported to elicit phototoxicity through the generation of reactive oxygen species following activation by exposure to visible or NIR light [33,34]. Moreover, the intrinsic fluorescence of GNCs makes them excellent contrast agents, owing to their high versatility and photostability [25,35,36,37].

The efficacy of a singular modality in breast cancer treatment could be impeded by multiple factors, including the intricate characteristics of the disease, the low bioavailability of therapeutic agents, the emergence of acquired resistance, etc. [8]. To overcome these limitations, combinational therapies have been developed to exploit the respective advantages of each modality [38,39]. In this study, a multifunctional nanocomposite system consisting of modified-GNRs, albumin-stabilized GNCs (BSA-GNCs, also referred to as GNCs in this manuscript), and doxorubicin (Doxo), was developed to be investigated for breast cancer treatment via combinational PTT and chemotherapy following a single dose of 808 nm NIR radiation. Firstly, GNRs were prepared with similar optical spectra and the conditions exhibiting the highest photothermal effect were selected. These GNRs were further modified with sodium citrate to increase their biocompatibility. Next, negatively charged GNCs were immobilized onto the citrate modified-GNRs via layer-by-layer assembly. The immobilized GNCs served as carriers for the chemotherapeutic agent Doxo and imparted the nanocomposite with intrinsic fluorescence for self-detection. Lastly, negatively charged hyaluronic acid (HA) was attached to the surface of the nanocomposite for targeting CD 44-overexpressed cancer cells. In vitro studies showed that the nanocomposite system remarkedly reduced cellular viability following the NIR irradiation.

## 2. Methods and Materials

Cetyltrimethylammonium bromide (CTAB, ≥98%) was obtained from Alfa Aesar (Blackfriars, MA, USA). Gold chloride trihydrate (HAuCl_4_·3H_2_O, ≥99.9%), silver nitrate (AgNO_3_, ≥99%), L-ascorbic acid (reagent grade), sodium oleate, sodium borohydride (NaBH_4_, ≥99.99%), glutaraldehyde solution (50 wt. % in water), dimethyl sulfoxide (DMSO), poly (sodium 4-styrenesulfonate) (PSS, 70 kDa), poly (allylamine hydrocholride) (PAH, 900,000), Chitosan (low molecular weight), and poly-l-lysine (PLL, 900,000) were purchased from Sigma–Aldrich, Saint Louis, MA, USA. Albumin bovine (BSA, biotechnology grade) was acquired from VWR. Hydrochloric acid (HCl, certified ACS plus) and sodium hydroxide (NaOH, 99.0%) were obtained from Fisher Scientific, Waltham, MA, USA. Hyaluronic acid (HA, 10,000) was bought from Lifecore Biomedical (Chaska, MN, USA). If not specified otherwise, all chemicals were prepared using Millipore water, Billerica, MA, USA.

### 2.1. GNR Synthesis

Three conditions were used to synthesize GNRs of different sizes following modified seed-mediated methods [40,41,42]. The seed solution and growth solution for each group were prepared separately in brand-new plastic tubes. All glassware was pre-cleaned with aqua regia and fully rinsed with Millipore water before use.

Group 1. Seed solution. HAuCl_4_ (2 mM, 625 µL) and water (1.35 mL) were added into CTAB (0.2 M, 1.88 mL), mixing well. NaBH_4_ (10 mM, 450 µL; freshly made in ice water) was quickly added into the gold solution under vigorous stirring. The seed solution was aged at 27 °C for 2–5 h. Growth solution. AgNO_3_ (10 mM, 500 µL) was added into bisurfactant solution (20 mL, 7 g CTAB and 1.234 g of sodium oleate were dissolved in 250 mL water, kept at 37 °C). The mixture was allowed to stand undisturbed for 15 min at room temperature before introducing HAuCl_4_ (2 mM, 10 mL) into the binary surfactant solution. The growth solution was shaken at 700 rpm for 90 min, after which HCl (12.1 M, 120 µL) was added. After shaking the growth solution at 400 rpm for an additional 15 min, L-ascorbic acid (0.1 M, 100 µL) was added and thoroughly mixed. Subsequently, a volume of 15 µL seed solution was added into the growth solution and left at 27 °C overnight. GNRs were collected by centrifugation at 10,000 rpm for 10 min. The sediment was resuspended in water for later use.

Group 2. Seed solution was prepared using the same method as mentioned above. Growth solution. HAuCl_4_ (1 mM, 25 mL) was introduced into CTAB (0.2 M, 25 mL) under stirring for 5 min. AgNO_3_ (0.1 M, 40 µL) and HCl (12 M, 40 µL) were added separately to the growth solution and mixed gently. L-ascorbic acid (0.1 M, 525 µL) was rapidly added into the stirring growth solution, resulting in an immediate change in solution color from brownish yellow to colorless. A volume of 250 µL seed solution was introduced and thoroughly mixed with the growth solution. The final mixture was kept at 27 °C overnight.

Group 3. Seed solution. The solution was prepared based on previous procedures [43,44]. HAuCl_4_ (10 mM, 0.5 mL) was added into CTAB (0.1 M, 9.5 mL) and stirred for 10 min. NaBH_4_ (10 mM, 710 µL, freshly made in 0.01 M of NaOH, ice cold) was quickly added to the gold solution with stirring at 1200 rpm and the solution turned into brown color immediately. The seed solution was stirred for 10 min before being kept at 27 °C for 2 h. Growth solution. HauCl_4_ (10 mM, 0.5 mL) was added into CTAB (0.1 M, 8 mL) under stirring for 10 min at 500 rpm. AgNO_3_ (100 mM, 40 µL) was introduced to the growth solution, followed by hydroquinone (0.1 M, 0.5 mL). The growth solution was gently mixed by inverting the tube between each step. After the growth solution turned colorless, a volume of 2 mL seed solution was introduced and mixed well. The resultant solution was left overnight at 27 °C. After which, GNRs were purified and collected through centrifugation at 13,200× *g* for 60 min. The sediment was dispersed in water for storage.

GNRs were characterized with UV-Vis-NIR spectrophotometry (UV 2600, Shimadzu, Kyoto, Japan), dynamic light scattering (Zeta sizer Nano ZS, Malvern, Worcestershire, UK), and scanning electron microscopy (SEM, Hitachi S 5500, Hitachi High-Technologies Corp, Tokyo, Japan).

### 2.2. Analysis of the Thermal Efficiency of GNR

GNRs (2 OD) from the three groups were digested in 300 µL of aqua regia (concentrated HCl: HNO_3_, 3:1) and left undisturbed for 2 h before adding the internal standard and diluting to 10 mL. Two microliters of 20 ppb Yttrium (TraceCEERT^®^) were added into each sample as the internal standard. To calculate the gold concentration in each sample, a standard linear curve was derived from a standard gold solution (TraceCEERT^®^). This curve was generated by plotting the response of working solutions against the known concentrations (0.1, 0.5, 1, 2, 3, 5, and 10 mg/L). The working solution and sample solution were, respectively, analyzed via inductively coupled plasma optical emission spectrometry (ICP-OES, Perkin Elmer, Waltham, MA, USA). Each analysis was performed in triplicate. The amount of gold in each sample was calculated via WinLab32 software.

To normalize the gold content in each group, medium-sized GNRs (group 2) were selected as the reference group. One milliliter of GNRs with the same absolute gold content from three groups were added into one 1.5 mL Eppendorf tube. An NIR laser (808 nm, 400 mW/cm^2^) was used to irradiate each tube for 10 min. The solution temperature was monitored with a thermal camera (Fluke, Everett, WA, USA, R3420) at 0, 1, 2, 3, 4, 5, 8, and 10 min. A curve depicting the relationship between the solution temperature and the radiation time was plotted. The GNRs group that exhibited the highest photothermal efficiency was selected for subsequent experiments.

### 2.3. GNR Modification

CTAB-capped GNRs were modified with biocompatible and versatile citrate ligand through PSS as the intermediary [45]. Briefly, GNRs (3 OD/mL) were incubated in 0.15% PSS for at least 1 h and centrifugated at 10,000 rpm for 40 min to obtain GNRs-PSS at the sediment; this incubation–centrifugation process was repeated at least twice to remove residual CTAB. Later, GNRs-PSS (3 OD/mL) were incubated in 5 mM of sodium citrate for at least 24 h and were collected by centrifugation to obtain citrate-stabilized GNRs as the sediment. To replace as much PSS as possible, citrate-GNRs (Cit-GNRs) were re-suspended in 5 mM of fresh sodium citrate and were incubated for another 24 h before centrifugation. The Cit-GNRs were dispersed in water and stored at 4 °C for later use.

Cit-GNRs (1, 2, and 4 OD/mL) were, respectively, added into 1.5 mL Eppendorf tubes. An NIR laser (808 nm, 400 mW/cm^2^) was used to irradiate each tube for 5 min, and a thermal camera (Fluke, R3420) was used to take the thermographic images at certain time intervals. The curves of solution temperature against the radiation time were plotted.

### 2.4. GNCs Synthesis

GNCs were synthesized using BSA as the template and reducing reagent [22]. Briefly, HAuCl_4_ (10 mM, 10 mL) was added into BSA aqueous solution (50 mg/mL, 10 mL) under vigorous stirring at 37 °C. After 5 min, NaOH (1 M, 1 mL) was introduced into the mixture, which was left to react for 12 h at 37 °C. The resulting GNCs were dialyzed against water for 48 h to remove impurities and stored at 4 °C for later use. For cellular experiments, the GNCs were lyophilized and diluted to specific concentrations with culture media.

The GNCs were characterized using a UV-vis-NIR spectrophotometer and a fluorescence spectrometer (PTI) with excitation at 470 nm. The gold amount in one milligram of BSA was detected using ICP-OES. Briefly, 200 µL of dialyzed GNCs was lyophilized and dissolved in 300 µL of aqua regia for 2 h. The resulting solution was diluted into 5 mL with water, and the gold concentration was calculated using the previously established method in Section 2.2.

### 2.5. Assembly of the Nanocomposite System

The Doxo base was prepared from doxorubicin hydrochloride following a protocol described previously [46]. Briefly, doxorubicin hydrochloride (50 mg) was dissolved in 5 mL of DMSO. Triethylamine (60 µL) was introduced into the DMSO mixture and stirred in the dark at room temperature for 12 h. The final solution was freeze-dried and desiccated.

A layer-by-layer assembly approach was used to construct the nanocomposite consisting of Cit-GNRs, GNCs, PAH, Doxo, and the targeting moiety HA. In the first step, negatively charged Cit-GNRs (3 OD/mL) were incubated in GNCs (10 mg/mL). After overnight incubation, the GNRs-GNCs composite was collected via centrifugation and then cross-linked with glutaraldehyde (GLA, 0.075%). In the second step, the composite from the first step (3 OD/mL) was introduced into a PAH solution (1 mg/mL) and incubated for 2 h to obtain the GNRs-GNCs-PAH composite, which were later incubated in GNCs (10 mg/mL) overnight. After the collection of the GNRs-GNCs-PAH-GNCs composite and a cross-linked process, Doxo (100 µg/mL) was added and incubated overnight in the dark, followed by the washing and collection of the GNRs-GNCs-PAH-GNCs-Doxo composite. In the third step, PAH (1 mg/mL) was incubated and deposited on the surface of the composite from step 2 to give it a positive charge, and then HA (10 mg/mL) was added for 10 min to create the final product GNRs-GNCs-PAH-GNCs-Doxo-PAH-HA. Composite with GNRs was centrifuged at 8000 rpm for 40 min after each deposition, and the surface charge and spectral absorption were monitored during this assembly process. The final product was kept in water at 4 °C until use.

Doxo in the supernatant was detected and calculated via the fluorescent intensity with a microreader (excitation 488 nm; emission 600/20 nm). The loading content of Doxo was calculated through the following formula: (µg. Doxo/OD. GNRs) = (Amount of added Doxo − Amount of Doxo in the supernatant)/GNR concentration. The preparation of rhodamine-labeled nanocomposite followed the same process, except that rhodamine 6G (5 µM) was substituted for Doxo. The final product was kept at 4 °C for later use.

### 2.6. Doxo Loading Efficiency and In Vitro Drug Release Profile

To investigate the release profile of Doxo, the nanocomposite system (10 OD) was added to a dialysis device with a molecular weight cut-off of 3500 Da (Slide-A-Lyzer, Thermos Scientific, Waltham, MA, USA). Subsequently, the dialysis device containing the nanocomposite was immersed in a tube with 10 mL of PBS (pH 7.4 or 5.5) as the release medium and maintained at 37 °C. At predetermined intervals of time (0.5, 1, 2, 4, 8, 12, 24, 48, 72, 96, and 120 h), 0.3 mL of release medium was withdrawn from each tube before an equal volume of PBS was added back to maintain the total volume. A calibration curve of Doxo (0.08, 0.16, 0.32, 0.63, and 1.25 µg/mL) against the corresponding fluorescent intensity was made via a microplate reader (excitation: 485/20 nm, emission: 600/40 nm). The released Doxo in PBS was detected using the calibration curve.

### 2.7. Cell Culture

Breast cancer MDA-MB-231 cells and human embryonic kidney 293 (HEK-293) cells were, respectively, maintained in McCocy’s 5A (ATCC) and DMEM medium (ATCC), containing 10% fetal bovine serum (ATCC) and 1% penicillin-streptomycin (Gibco, Carlsbad, CA, USA) at 37 °C in 5% CO_2_, unless otherwise mentioned.

### 2.8. Cellular Toxicity

MTS (3-(4,5-dimethylthiazol-2-yl)-5-3-carbomethoxyphenyl)-2-(4-sulfophenyl)-2H-tetrazolium) experiment was performed to evaluate the cellular toxicity in vitro. MDA-MB-231 cells were seeded at the density of 30,000 cells per well in a 96-well plate. CTAB-capped GNRs, Cit-GNR, and nanocomposite without Doxo (1 OD/mL) were, respectively, added into cells and incubated for 24, 48, or 72 h. At the end of each time interval, the old medium was replaced with 100 µL of PBS containing 20 µL of MTS working solution and incubated for 4 h in an incubator. The MTS working solution was a mixture of MTS solution and phenazine methosulfate (PMS) solution in a ratio of 20:1. The optical density at 490 nm was determined. Cells incubated in culture media were only used as the control group.

### 2.9. Cellular Uptake

To assess the cellular uptake of the nanocomposite with minimal auto-fluorescence, rhodamine 6G was integrated into the nanocomposite. MDA-MB-231 cells were seeded in glass bottom 6-well plates at a density of 20,000 cells per well. The rhodamine-labeled nanocomposite (1 OD/mL) suspended in medium was added to the cells and incubated for 24 h. Following three washes with PBS, Hoechst 33324 (8 µM, 200 µL) was applied to stain the cellular nuclei. The cellular uptake of the nanocomposites was observed using a confocal microscope (Leica, DMi8, Wetzlar, Germany). The cellular uptake efficiency was analyzed based on fluorescence after 24 h incubation. The uptake experiment was also performed on HEK-293 cells to evaluate the affinity of the nanocomposite for cells with low or no CD44 receptors. The emission of stained nuclei and nanocomposite was measured at 420–480 nm and 550–720 nm, respectively.

### 2.10. Photothermal Therapy In Vitro

MDA-MB-231 cells were seeded at a density of 30,000 cells per well in a 96-well plate and incubated overnight. Nanocomposites (0.25 and 0.5 OD/mL) were added into the cells. After incubation for 6 h, the nanocomposite-treated cells were subjected to laser irradiation (808 nm, 400 W/cm^2^) for 4 min. The MTS assay was performed at 24, 48, and 72 h to access the cellular viability of the nanocomposite-treated cells compared to untreated cells.

### 2.11. Three-Dimensional Cellular Culture

To overcome the limitations of two-dimensional cellular cultures, a three-dimensional model was established to better simulate the architecture, heterogeneity, and complexity of tumors. MDA-MB-231 cells were seeded at a density of 1000 cells per well in 100 µL of media in a Corning^®^ spheroid microplate (Ref 4515). After 24 h, 50 µL of the old medium was removed from each well, and 50 µL of fresh medium containing 2% Matrigel (BD science, Franklin Lakes, NJ, USA) was added. The plate was then gently shaken at 120 rpm for 10 min to promote spheroid formation. Single compact spheroids were allowed to form overnight, establishing day 0 of this experiment. Subsequently, nanocomposites (0.25 and 0.5 OD/mL) were added to the cellular spheroids and incubated for 5 days. On Day 1, photothermal therapy was performed using an 808 nm laser (400 mM/cm^2^, 4 min). Light microscopy was used to capture images of the spheroids at both bright field and dark field throughout the experimental period. The cellular viability of the spheroids was assessed using the Cell Titer-Glo^®^ 3D cell viability assay (Promega, Madison, WI, USA) on Day 3 and Day 5.

### 2.12. Statistical Analysis

One-way analysis of variance (ANOVA) was conducted to determine the significant difference among the multiple groups using GraphPad Prism 8. *p*-values lower than 0.05 were defined to be statistically significant.

## 3. Results and Discussion

### 3.1. Synthesis and Characterization of GNRs

**Figure 1:**
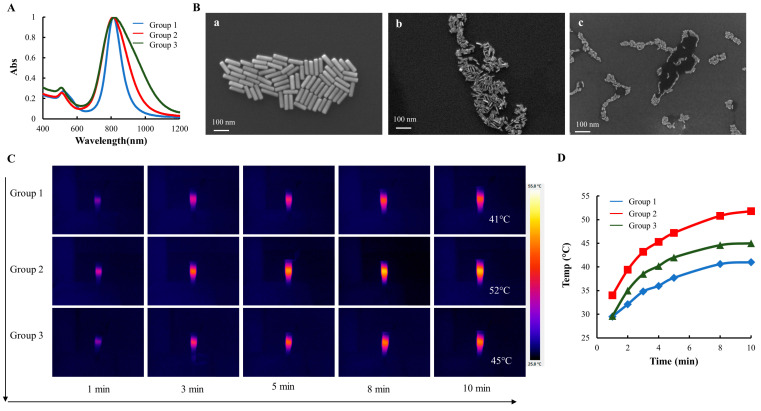
GNR Characterization. (**A**) UV-Vis-NIR spectra of GNRs (blue curve for group 1, red curve for group 2, and green curve for group 3). (**B**) SEM images of GNRs: (**a**) for group 1, (**b**) for group 2, and (**c**) for group 3). (**C**) Thermographic images of three groups of GNRs over 10 min. (**D**). The plot of temperature over time following the NIR radiation (400 mW/cm^2^).

It is well-established that the SPR of GNRs can be tuned by adjusting their aspect ratio, i.e., the ratio of the nanorod’s length to its width or diameter. Enhanced photothermal conversion can be achieved by adjusting the SPR wavelength to match the wavelength of incident light. To investigate the impact of GNR size on the photothermal efficiency, three distinct sets of GNRs exhibiting similar SPR spectra, with wavelengths at approximately 808 nm, were synthesized. As shown in Figure 1A, the spectral curves of all three groups of GNRs had narrow and smooth distributions, indicating that there was minimal colloidal aggregation. The UV-Vis-NIR spectra of GNRs have two distinct SPR peaks because of their anisotropic shape. Specific to this batch of GNRs, TSPR peaks for the three groups were located at 520 nm, 509 nm, and 506 nm, while LSPR peaks were located at 813 nm, 812 nm, and 800 nm, respectively. The TSPR peak location is positively correlated with the transverse diameter of GNRs, with thinner GNRs having relatively small TSPR. As the diameter increases, the TSPR peak shifts to the right of the UV-Vis-NIR spectrum. The LSPR peak, on the other hand, is correlated with the aspect ratio, i.e., the ratio of the length to the diameter of the GNRs.

SEM images of the three groups of GNRs were taken to visualize their morphology (Figure 1B). The effective sizes of each group of GNRs were calculated based on ~150 randomly selected GNRs (Table 1). The spectral absorption curves and SEM images showed that thick GNRs (group 1) had the narrowest size distribution, while thin GNRs (group 3) had the widest spectral absorption. GNRs (group 2) with a mean diameter of ~9 nm was in between, as shown in Figure 1A.

The gold content measurements for the three groups were as follows: 10.3 ± 0.28 µg/OD for group 1, 9.1 ± 0.25 µg/OD for group 2, and 10.9 ± 0.40 µg/OD for group 3. In order to normalize the gold content across the groups, medium-sized GNRs from group 2 were chosen as the reference. Consequently, 1 mL of 0.88 OD/mL GNRs from group 1, 1 OD/mL GNRs from group 2, and 0.84 OD/mL GNRs from group 3 were used to investigate their photothermal properties. Thermographic images of GNRs exposed to the NIR radiation (400 mW/cm^2^) for a range of 1–10 min were captured and presented using a color spectrum, where blue represents low temperatures and a bright yellow color represents higher temperatures (Figure 1C). The dynamic temperature profile revealed that the temperature of the GNRs solution increased with longer radiation time, and higher GNR concentrations resulted in increased heat generation and higher temperature levels (Figure 1D). Notably, GNRs from group 2 showed the fastest increase in the solution temperature and reached the highest final temperature (52 °C). Since the GNRs from group 2 exhibited the most efficient photothermal effect, they were selected for subsequent experiments.

**Figure 2:**
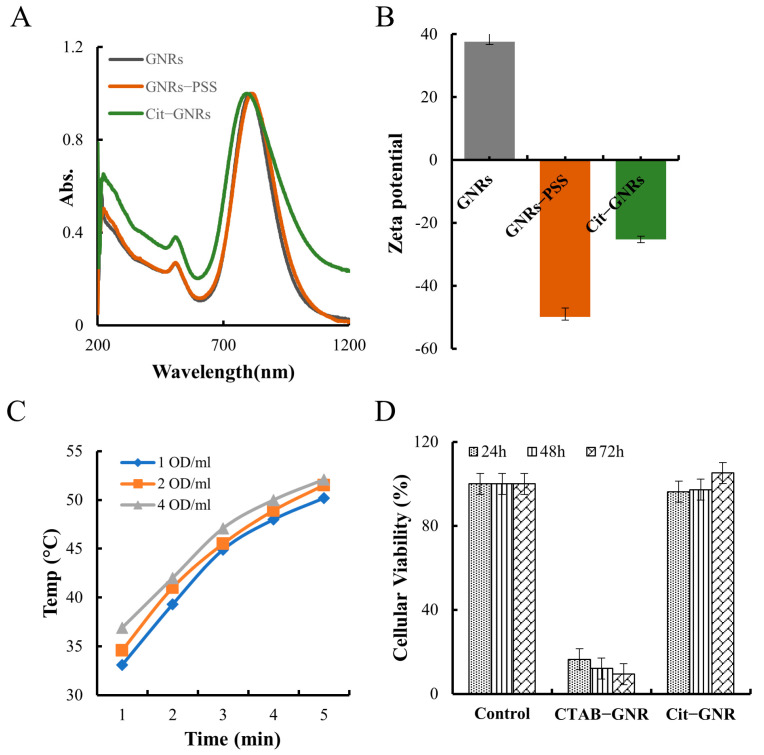
GNRs modification and characterization. (**A**) UV-vis spectra of CTAB-GNRs, GNR-PSS, and Cit-GNRs. (**B**) Zeta potential of CTAB-GNRs, GNR-PSS, and Cit-GNRs. (**C**) The plots of solution temperature of GNRs (1, 2, and 4 OD/mL) over 5 min. (**D**) Cellular toxicity result of CTAB-GNR and Cit-GNRs (1 OD/mL).

In GNR synthesis, CTAB serves as both the template and stabilizer. However, CTAB-capped GNRs can bind to cell membranes, which may inadvertently cause cell death due to nonspecific electrostatic interactions with negatively charged cell surfaces [47]. To mitigate the negative impact of CTAB and provide a temporary stabilization for GNRs, PSS was employed as an intermediate stabilizing agent [45]. As Figure 2A,B show, the LSPR peak of GNRs-PSS colloids shifted 8–10 nm to the red side after PSS absorption; the surface potential was reversed from 30 ± 5 mV (GNRs) to −50 ± 3 mV (GNRs-PSS). Polyanions-sodium citrate was then used to replace PSS on the surface of GNRs via ligand exchange. This exchange resulted in a blue shift of the LSPR peak of Cit-GNRs by 8–10 nm (Figure 2A) and its surface potential was −25 ± 3 mV (Figure 2B) and less negative compared with GNRs-PSS [45]. The spectral profiles of GNRs and Cit-GNRs were similar, as shown in Figure 2A, suggesting that the citrate ligand exchange process had no discernible impact on the GNRs’ shape or size. It is important to note that PSS played a critical role in the GNRs modification, as the direct addition of sodium citrate into CTAB-capped GNRs would lead to irreversible aggregation. By introducing PSS as an intermediary, sodium citrate was able to displace CTAB, render good colloidal stability to the Cit-GNRs, and confer enhanced versatility and adaptability for their surface modification and ligand exchange. Sodium citrate is a surface-active agent, and its citrate anions can offer moderate binding affinity to Au present on the GNRs’ surface [37].

The photothermal effect of Cit-GNRs (1, 2, and 4 OD/mL) was evaluated using laser radiation (400 mW/cm^2^, 5 min). Regardless of concentration, there was a positive relationship between the radiation time and the solution temperature (Figure 2C). Moreover, higher concentrations of GNRs generated more heat and higher temperatures. Notably, at all three tested concentrations, the solution temperature surpassed 42 °C by the third minute of radiation. As shown in Figure 1D and Figure 2C, the surface coating of GNRs, whether it be citrate or CTAB, did not affect the GNRs photothermal effect.

An MTS experiment was performed to determine the toxicity of CTAB-capped GNRs (1 OD/mL) and Cit-GNRs (1 OD/mL) on MDA-MB-231 cells. The cellular viability of the CATB-GNRs was 16% after 24 h and further reduced to 6% at 72 h; on the contrary, the cellular viability of the Cit-GNRs was comparable to those of the control group at all time intervals (Figure 2D). Positively charged CTAB-capped GNRs can undergo nonspecific electrostatic interactions with negatively charged biomolecules or cellular membranes. Such nonspecific interactions primarily caused the cell death in the CATB-GNR group [47]. However, when CTAB was replaced with citrate, the negatively charged Cit-GNRs showed no significant adverse effects on cellular viability.

### 3.2. Synthesis and Characterization of GNCs

**Figure 3:**
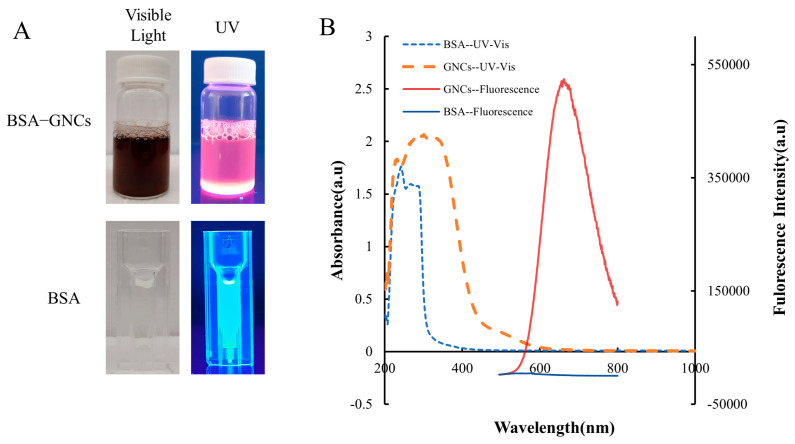
BSA-GNCs characterization. (**A**) Images of BSA-GNCs and BSA under visible light and UV radiation. (**B**) Optical and fluorescent spectra of BSA and BSA-GNCs (excitation/emission: 470 nm/642 nm).

BSA, a ubiquitous blood plasma component, has a range of applications in pharmaceutical and biomedical fields owing to its high biocompatibility, biodegradability, low or non-immunogenicity, versatility, and affordability [48]. In this study, the GNCs were synthesized and stabilized using BSA as the template. The BSA molecules acted as chelating agents, sequestering Au (III) ions and entrapping them. The trapped gold ions were subsequently reduced to Au (0) in a high pH solution and protected by thiol groups from BSA cysteine residues [22]. The solution of BSA-stabilized GNCs appears brown under visible light and emits bright red fluorescence when exposed to UV light at 302 nm; the BSA solution is colorless under visible light and exhibits blue fluorescence when illuminated with UV light at 302 nm (Figure 3A). The fluorescence and UV-Vis spectra of BSA and GNCs were also investigated, with notable differences in their peaks (Figure 3B). The ICP-OES result showed that there was approximately 39 ± 0.3 µg of gold per milligram of BSA.

### 3.3. Assembly of the Nanocomposite System

The layer-by-layer adsorption technique provides versatility in material selection and structural design, making it suitable for drug delivery systems that require complex fabrication requirements. The utilization of the layer-by-layer method has proven to be a simple and efficient method for functionalizing GNRs due to their diverse surface chemistry. A layer of GNCs was initially coated onto Cit-GNRs. The presence of amino acid residues in BSA-stabilized GNCs provided ample sites for conjugation with GNRs [49]. Glutaraldehyde, an effective protein cross-linking reagent, was employed to facilitate the crosslinking and immobilization of GNCs stabilized by BSA. The crosslinking process offered two advantages: enhancing the stability of the nanocomposite and providing binding sites for Doxo molecules. PAH, a cationic electrolytic polymer, was introduced to reverse the surface potential and increase the accumulation of GNCs on the surface of GNRs. Additionally, a layer of hyaluronic acid was coated on the exterior, serving as a targeting ligand.

**Figure 4:**
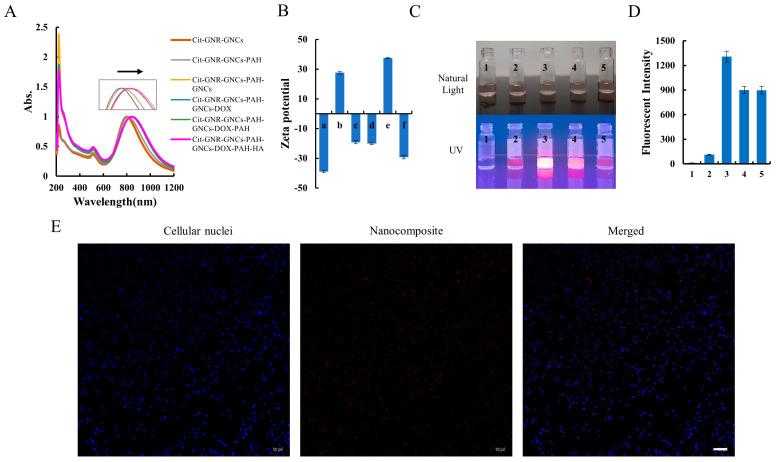
Assembly of the nanocomposite with a layer-by-layer approach. (**A**) Sequential optical spectra of GNRs colloidal, with LSPR shift to red along with each deposition. (**B**) Zeta potential of GNRs with different depositions (a. Cit-GNRs-GNC; b. Cit-GNRs-GNC-PAH; c. Cit-GNRs-GNC-PAH-GNCs; d. Cit-GNRs-GNC-PAH-GNCs-Doxo; e. Cit-GNRs-GNC-PAH-GNCs-Doxo-PAH; f. Cit-GNRs-GNC-PAH-GNCs-Doxo-PAH-HA). (**C**) Images of GNRs with different depositions (1. Cit-GNRs; 2. Cit-GNRs-GNC; 3. Cit-GNRs-GNC-PAH-GNCs; 4. Cit-GNRs-GNC-PAH-GNCs-Doxo-PAH; 5. Cit-GNRs-GNC-PAH-GNCs-Doxo-PAH-HA) under visible light and 302 nm UV radiation. (**D**) Fluorescent intensity of GNRs with different depositions in panel C under a microreader excited at 485 nm and emission at 640 nm. (**E**) The image of MDA-MB-231 cells incubated the nanocomposite (no Doxo) for 24 h; magnification 10×. scale bar, 100 µm.

The success of each deposition step was confirmed by observing zeta potential reversal and SPR peak shift, as these indicators are sensitive to changes in the dielectric environment, as well as colloidal stability and aggregation. Following each deposition step, a spectral shift of a few nanometers was observed in the spectra (Figure 4A). The SPR peak was shifted from 801 ± 3 nm (Cit-GNRs-GNCs) to 815 ± 3 nm (Cit-GNRs-GNCs-PAH), 820 ± 2 nm (Cit-GNRs-GNCs-PAH-GNCs), 827 ± 2 nm (Cit-GNRs-GNCs-PAH-GNCs-Doxo), 840 ± 2 nm (Cit-GNRs-GNCs-PAH-GNCs-Doxo-PAH), and 845 ± 3 nm (Cit-GNRs-GNCs-PAH-GNCs-Doxo-PAH-HA), respectively. However, the spectral shape of SPR peaks remained unchanged, indicating the absence of noticeable aggregation during the layer-by-layer assembly process. In parallel, zeta potential values exhibited alternate reversals with each deposition step, except for the initial layer of GNCs (Figure 4B). The surface charge of Cit-GNRs was negative, primarily attributed to the presence of chloride and citrate ligands. However, upon introducing BSA-GNCs, the zeta potential of Cit-GNRs-GNCs decreased further from −25 ± 2 mV to −39 ± 1 mV (Figure 2B and Figure 4B). This decrease was indicated by a stronger negative surface charge resulting from the deposition of GNCs. The self-assembly also involves other interactions, such as electrostatic, hydrogen bonds, acid-base and coordination interactions, or even covalent bonds [50]. The cross-linking with GLA did not cause any spectral shift or surface charge change (Figure S1). The peak at ~250 nm disappeared after the GLA cross-linkage, implying the success of cross-linking.

When illuminated under a UV transilluminator at 302 nm, Cit-GNRs colloids (1 OD/mL) with varying deposition layers displayed differences in fluorescence intensities, despite appearing similar in color under visible light (Figure 4C,D). Among the different deposition layers, GNRs with two layers of GNCs exhibited the highest fluorescence intensity, followed by GNRs with an additional layer of both PAH and HA, and then by GNRs with just a single layer of GNCs. In this study, a layer-by-layer approach was adopted to attach GNCs onto Cit-GNRs through electrostatic bonds. However, these bonds might be relatively weak, leading some outer GNCs to be washed away in the later step. This vulnerability in the attachment process could explain why Cit-GNRs with two layers of GNCs emitted the strongest fluorescence. Additionally, it is noteworthy that the fluorescent intensity of Cit-GNRs with an additional layer of both PAH and HA remained consistent (Figure 4D).

To further investigate the fluorescence of the nanocomposite in cells, 1 OD/mL of the nanocomposite was added into MDA-MB-231 cells and incubated for 24 h. As demonstrated in Figure 4E, the nanocomposite fluorescence facilitated the localization and identification of nanocomposite within cells.

### 3.4. Doxo Loading Efficiency, Release Profile, and the Stability of the Nanocomposite System

Doxo, a wide-spectrum anti-cancer drug, was selected as a model drug due to its favorable physiochemical properties for formulation research. It was efficiently loaded onto the second layer of GNCs via electrostatic binding with the BSA residues. The loading content was calculated, and the loading amount was 0.71 ± 0.15 µg. Doxo/OD. GNRs, or 1.31 ± 0.28 µM. Doxo/OD. GNRs.

**Figure 5:**
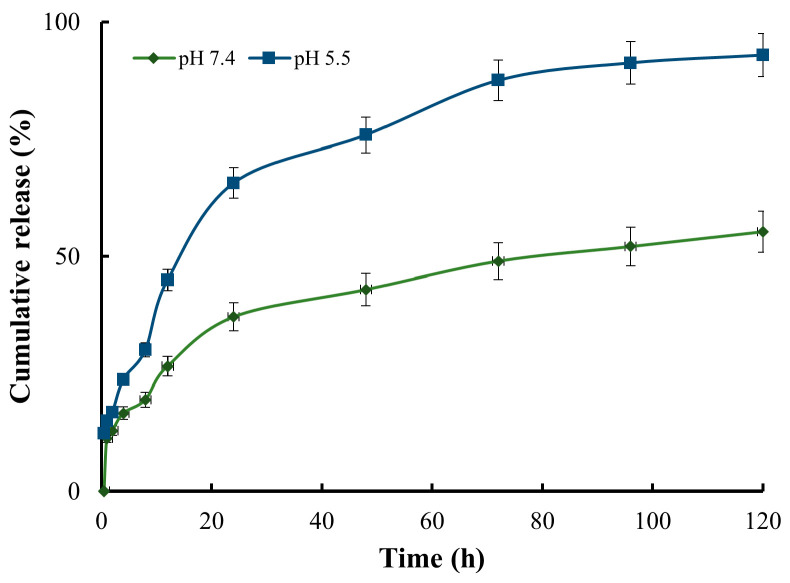
The release profile of Doxo from the nanocomposite in different pH environments (5.5 and 7.4) within 120 h.

Insufficient oxygen supply and a build-up of metabolic wastes result in the tumor microenvironment becoming hypoxic and acidic, which incentivizes the design of pH-sensitive drug delivery systems [51,52]. To investigate the pH sensitivity of the nanocomposite system, Doxo release profile was measured in two different pH environments: 7.4, which represents normal physiological conditions, and 5.5, which represents an acidic tumor site. At pH 7.4, the release of Doxo was significantly slow, with only 37% released within 24 h. Throughout the entire 120 h study, a total of 55% of Doxo was released. In contrast, Doxo exhibited rapid release at pH 5.5: approximately 65% of Doxo was released within 24 h, over 85% within 72 h, and more than 95% within 120 h (Figure 5). These results indicated that Doxo was released from the nanocomposite in a pH-dependent way, as the cross-linked bonds formed by GLA were reversible under an acidic environment and could expedite the diffusion of Doxo. This structural characteristic is advantageous for tumor drug delivery as Doxo can be securely entrapped and protected within the nanocomposite under normal physiological conditions while experiencing rapid release in an acidic environment, a hallmark of tumor sites.

### 3.5. Cellular Uptake Assay

**Figure 6:**
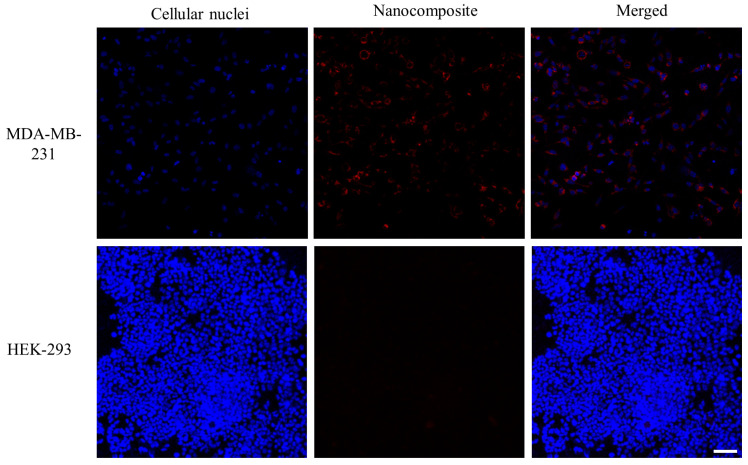
Cellular uptake of the nanocomposite in MDA-MB-231 and HEK-293 cells within 24 h. The cellular nuclei were stained with Hoechst 33324 to locate cells. Nanocomposite was labeled with Rhodamine 6G. Magnification 20×; scale bar, 50 µm.

The cellular uptake of the nanocomposite was investigated in MDA-MB-231 and HEK-293 cells. Following 24 h of incubation, the fluorescent images of cellular nuclei (blue) stained with Hoechst 33324 and nanocomposites (red) labeled with Rhodamine 6G were taken under a confocal microscope. By merging these images, the location of the nanocomposite can be pinpointed with the cell. A significantly low red fluorescent intensity was detected in the cellular plasm of HEK-293 cells, suggesting minimal internalization of the nanocomposite. In contrast, MDA-MB-231 cells exhibited strong red fluorescence, indicating a robust cellular uptake of the nanocomposite (Figure 6). The disparity in cellular uptake between MDA-MB-231 and HEK-293 cells can be attributed to the presence of CD44 receptors, which are common to MDA-MB-231 and have a high affinity for the HA-targeting ligands of the nanocomposite.

### 3.6. Treatment Efficiency of the Nanocomposite In Vitro

GNRs exhibit high thermal conversion efficiency, which makes them effective photothermal agents. When GNRs absorb radiant energy, they convert the absorbed radiation into heat, which can lead to cellular dysfunctions in surrounding cells and tissues. Although NIR radiation is considered low-energy and generally safe for normal cells and tissues, it can still potentially induce non-thermal DNA damage [53]. To ensure the safety and effectiveness of the combinational therapy with NIR irradiation in this study, the optimal photothermal parameters, including GNRs concentration and irradiation time, for cellular experiments were investigated.

Four concentrations of Cit-GNRs (0.125, 0.25, 0.5, and 1 OD/mL) suspended in the cell culture media were tested. Figure S2A,B demonstrated that the concentration of 0.125 OD/mL did not achieve the required temperature for effective thermal treatment. Concentrations of 0.25 and 0.5 OD/mL of Cit-GNRs increased the temperature up to 37 °C at 3 min and above 40 °C at 4 min, while the concentration of 1 OD/mL reached 45 °C within 2 min. Based on these results, the three concentrations that achieved the threshold temperature (0.25, 0.5, and 1 OD/mL) and an irradiation dosage of 400 mW/cm^2^ for 4 min, were used as the photothermal parameters for subsequent cellular experiments.

MDA-MB-231 cells were treated with the three different concentrations of Cit-GNRs, followed by photothermal treatment at 400 mW/cm^2^ for 4 min. The result indicated that, after 72 h, the cellular viability in the groups treated with 0.25 and 0.5 OD/mL of Cit-GNRs remained above 95% and comparable to the untreated group (Figure S2C). However, in the 1 OD/mL group, cellular viability significantly decreased from 24 h, reaching only 15% at 72 h. Notably, the photothermal irradiation alone did not negatively impact cell viability. As a result, the concentrations of 0.25 and 0.5 OD/mL were selected for further cellular experiments involving combinational photothermal therapy.

**Figure 7:**
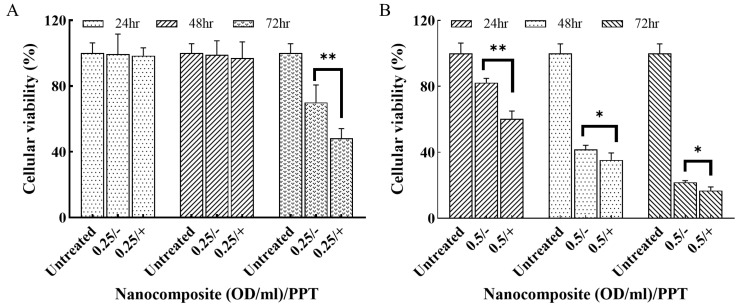
The cellular viability of MDA-MB-231 cells treated with the nanocomposite with/o PTT. (**A**) 0.25 OD/mL of the nanocomposite. (**B**) 0.5 OD/mL of nanocomposite. “+” refers to PTT and “−” refers to no PTT. * *p* < 0.05, ** *p* < 0.001.

The treatment efficacy of the nanocomposite at the concentrations of 0.25 and 0.5 OD/mL with and without PTT was assessed using MDA-MB-231 cells. After 72 h treatment, the cellular viabilities of the groups, i.e., 0.25/−, 0.25/+, 0.5/−, and 0.5/+, were 90%, 48%, 21%, and 16%, respectively. Notably, the cellular viability in the combination treatment groups (Doxo + PTT) were significantly lower than the single-modality groups (Doxo only) for both concentrations (Figure 7).

### 3.7. In Vitro Treatment Efficiency on 3D Cell Culture

**Figure 8:**
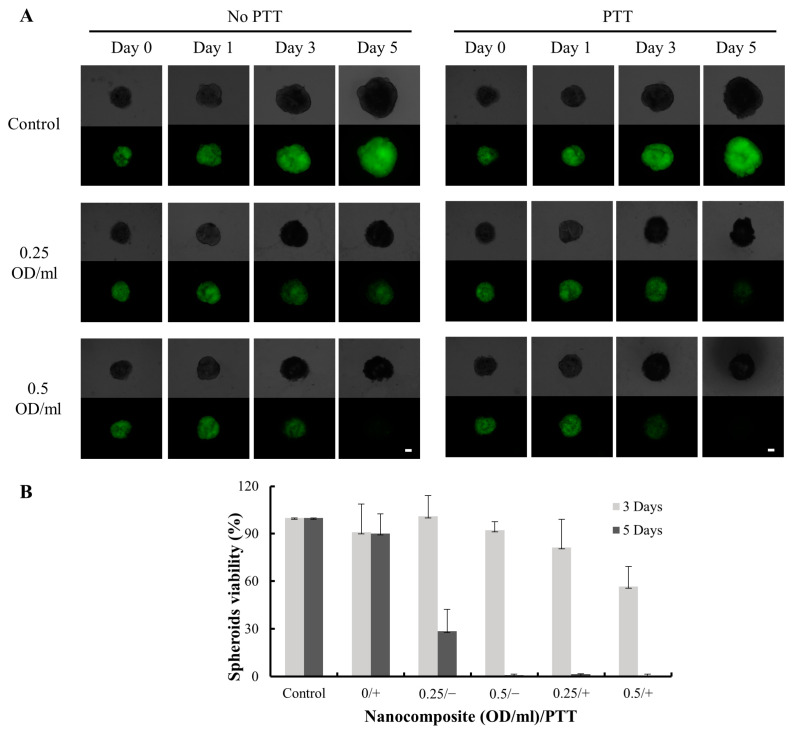
Cellular viability of MDA-MB-231 cells treated with the nanocomposite and PTT. (**A**) Brightfield (upper half) and darkfield (lower half) images of 3D spheroidal under fluorescent microscopy on Day 0, 1, 3, and 5. The scale bar is 100 µm. (**B**) The cellular viability of spheroids was analyzed on Day 3 and Day 5. “+” = PTT; “−” = no PTT.

The viability of spheroids was assessed for 3- and 5-day incubation periods. On Day 3, the cellular viability of the non-PTT group was 99% at the concentration of 0.25 OD/mL, while the PTT treatment group showed viability of 81%, resulting in a 17% decrease in cellular viability due to PTT (Figure 8). At the higher concentration of 0.5 OD/mL, the cellular viability was 92% for the non-PTT group and 57% for the PTT treatment group, representing a 35% reduction in viability due to PTT treatment. On Day 5, a highly significant difference was observed between the PTT group (with less than 5% cellular viability) and the non-PTT group (with 29% cellular viability) at the concentration of 0.25 OD/mL. In the higher concentration group, most cells were dead irrespective of PTT treatment. When compared with the untreated group, nanocomposite treatment significantly reduced cellular viability, both with and without PTT. The nanocomposite exhibited inhibitory effects on cellular viability by releasing Doxo and performing chemotherapy even in the absence of PTT. However, the efficacy of the nanocomposite was significantly increased with PTT treatment at both 0.25 and 0.5 OD/mL concentrations. PTT enhanced the therapeutic effect and sensitivity of Doxo, consistent with the findings observed in the 2D cell culture system.

## 4. Conclusions

GNRs have distinct optical properties, allowing for efficient manipulation of their photothermal performance through remote control using NIR radiation. The NIR energy within the range of 650 to 950 nm can easily penetrate tissue with minimal scattering, while biomacromolecules exhibit minimal absorption of NIR light [17,54]. This enables the targeted delivery of NIR energy to GNRs embedded within specific issues without causing harm to healthy tissue. These combined characteristics position GNRs as promising candidates for photothermal applications. However, the efficiency of GNRs in absorbing and scattering light is dependent on their diameters. This study compared three groups of GNRs with highly similar optical spectra but varying diameters (~5 nm, ~9 nm, and ~28 nm). Among them, the group with an average diameter of ~9 nm demonstrated the most effective photothermal effect. This discovery emphasizes a key relationship between GNRs size and photothermal performance.

To enhance the functionality of GNRs, BSA-conjugated GNCs were successfully assembled onto GNRs using the layer-by-layer technique, resulting in a nanocomposite with fluorescence and self-labeling capabilities. The residual BSA molecules also provided binding sites for Doxo. This study used Doxo as a model drug, but this nanocomposite is generalized enough such that Doxo can be substituted with other therapeutic or diagnostic agents. The targeting moiety, HA, facilitated high cellular internalization and specificity for MDA-MB-231 cells due to their overexpression of CD44 receptors. The nanocomposite exhibited stability in a physiological environment but triggered Doxo release in acidic environments or following NIR radiation.

Cellular viability served as a measure to assess therapeutic efficacy in 2D and 3D cell culture systems. The nanocomposite exhibited a notable decrease in cellular viability and demonstrated effective therapy. Additionally, the implementation of photothermal therapy significantly enhanced the therapeutic effect of chemotherapy. The combination therapy displayed superior inhibitory effects on both monolayer cancer cells and spheroids.

In summary, we developed a robust nanocomposite consisting of GNRs and GNCs as a potential system for breast cancer treatment. The layer-by-layer assembly technique offers simplicity and reproducibility, allowing for control over GNC layering and drug loading for specific applications. The straightforward synthesis, simple conjugation chemistry, and cost efficiency of this delivery system make it a versatile platform which can be readily adapted to explore the therapeutic efficacy of different drugs, varied GNCs layers, and combinations of photothermal therapy with minimal modifications.

## Figures and Tables

**Table 1:** Actual sizes and gold content of GNRs in each group.

Group	Group 1	Group 2	Group 3
Size (length × diameter), nm	96.4 ± 10.2 × 28.2 ± 3.4	31.8 ± 7.3 × 8.6 ± 1.7	19.1 ± 2.4 × 4.8 ± 0.3
Absolute gold content, µg/OD	10.3 ± 0.28	9.1 ± 0.25	10.9 ± 0.40

## Data Availability

Not applicable.

## Supplementary Materials

The following supporting information can be downloaded at: https://www.mdpi.com/article/10.3390/pharmaceutics15092198/s1, Experimental for polymer screening; cross-link of GNRs-GNCs; the photothermal effect of citrate-stabilized GNRs; and the stability of the nanocomposites. Table S1: The quantity of GNCs assembled onto GNRs; Figure S1: Optical spectra and zeta potential of GNRs-GNCs before and after GLA cross-link; Figure S2: Thermographic images and temperature change in Cit-GNR over time under laser illumination (808 nm, 400 mW/cm^2^); and Figure S3: The optical spectra of nanocomposite storage at 4 °C for 3 months.
