# Supplementary material for: Gold Nanomaterial System That Enables Dual Photothermal and Chemotherapy for Breast Cancer
Source: Pharmaceutics. 2023 Aug 25;15(9):2198. doi: 10.3390/pharmaceutics15092198 (PMC10534904; doi:10.3390/pharmaceutics15092198)
Supplement: Supplementary file 1 [file pharmaceutics-15-02198-s001.zip › pharmaceutics-2564729-supplementary.pdf]

Gold Nanomaterial System that Enables Dual Photothermal and Chemotherapy for Breast Cancer

Lijun Wang [1], Binita Shrestha [2], Eric M. Brey [1,*] and Liang Tang [1,*]

1 Department of Biomedical Engineering & Chemical Engineering, University of Texas at San Antonio, San Antonio, TX 78249, USA

2 Department of Biomedical Engineering, University of Texas at Austin, Austin, TX 78705, USA

* Correspondence: eric.brey@utsa.edu (E.M.B.); liang.tang@utsa.edu (L.T.)

1. Method

Cetyltrimethylammonium bromide (CTAB, ≥98%) was obtained from Alfa Aesar. Gold chloride trihydrate ($HAuCl_4 \cdot 3H_2O$, ≥99.9%), silver nitrate ($AgNO_3$, ≥99%), L-ascorbic acid (reagent grade), sodium oleate, sodium borohydride ($NaBH_4$, ≥99.99%), glutaraldehyde solution (50 wt. % in water), poly (sodium 4-styrenesulfonate) (PSS, 70kDa), poly (allylamine hydrocholride) (PAH, 900,000), Chitosan (low molecular weight), and poly-l-lysine (PLL, 900,000) were purchased from Sigma-Aldrich. Albumin bovine (BSA, biotechnology grade) was acquired from VWR. Hydrochloric acid (HCl, certified ACS plus) and sodium hydroxide (NaOH, 99.0%) were obtained from Fisher Scientific. Hyaluronic acid (HA, 10,000) was bought from Lifecore Biomedical (Chaska, MN). If not specified otherwise, all chemicals were prepared using Millipore water.

1.1 Polymer Screening

Three polymers, PLL, chitosan, and PAH, were examined and evaluated for their affinity toward GNCs. The screening procedure involved employing the layer-by-layer technique as outlined in the subsequent section. Once two layers of GNCs were deposited on the surface of Cit-GNRs, the quantity of GNCs was determined using a NanoDrop and UV-Vis-NIR spectrophotometry. These two instruments were utilized separately to quantify the concentrations of GNCs and Cit-GNR in the nanocomposite. Since GNCs have negligible absorption in the Vis-NIR range, the concentration of Cit-GNRs can be determined by measuring the optical absorbance of the nanocomposite (Cit-GNRs-GNCs-GNCs). By utilizing the BSA module in the NanoDrop, the absorbance of nanocomposite can be measured, and by knowing the concentration of Cit-GNRs, the specific absorbance contributed by Cit-GNRs can be calculated. Here are the detailed steps:

Firstly, the concentration of a Cit-GNR stock solution was determined by analyzing its UV-Vis-NIR spectrum, specifically by measuring the height of the LSPR peak. The absorbance of Cit-GNRs (0.32, 0.65, 1.3, 2.6, 5.3, 10.5, and 21 OD/ml) was measured using the BSA module in the Nanodrop. A linear curve was generated by plotting the known Cit-GNR concentrations against their absorbance measured with the Nanodrop (Plot A). Secondly, the absorbance of GNCs (based on BSA, 0.5, 1, 2, 5, 8, and 10 mg/ml) was also measured using the NanoDrop. A plot of GNCs concentrations against their absorbance was made (Plot B). Finally, the concentration of Cit-GNRs in the nanocomposite solution was determined using the UV-Vis-NIR and referred to as C_{GNR}, so the absorbance attributed to the GNRs contribution (A_{GNR}) could be calculated using Plot A. The absorbance of the nanocomposite was measured using the NanoDrop and referred to as A nanocomposite. The following formula was used to estimate the amount of GNCs: GNCs (mg/OD) = $(A_{nanocomposite} - A_{GNR})/C_{GNR}$. It should be noted that this method cannot provide an exact measurement of the GNCs quantity on GNRs; however, the calculated value does reflect the GNCs assembly and was applied to screen different polymers and select an appropriate polymer for further study.

1.2 Cross-link of GNRs-GNCs

Glutaraldehyde (GLA) was gradually added to the GNRs-GNCs colloidal solution (1 OD/ml) under stirring at 400rpm, making the final concentration of GLA at 0.075%. The cross-linking reaction was conducted for 12 h at room temperature, after which the colloids were collected by centrifugation. The optical spectrum and zeta potential of GNRs-GNCs were detected both before and after the cross-link process.

1.3 Photothermal effect of Cit-GNRs

Aliquot of 200 µl of Cit-GNRs (0.125, 0.25, 0.5 and 1 OD/ml) suspended in medium were added into a 96-well plate. Each well was then irradiated with an 808 nm NIR laser (400 mW/cm^2) for 5 min.

During the NIR irradiation, a thermal camera was used to take thermographic images of each well and monitor the temperature change, allowing for the construction of a plot illustrating the temperature.

MDA-MB-231 cells were seeded at a density of 30,000 cells per well and cultured in 96-well plate overnight. Cit-GNRs (0.25, 0.5 and 1 OD/ml) suspended in 200 µl of medium were added into cells. After incubation with Cit-GNRs for 6 h, cells were radiated with 808nm NIR (400 mW/cm^2, 4 min) and continued to be incubated until the cellular viability assay at 24, 48, or 72 h.

1.4 Stability

The physical stability of the nanocomposite was assessed under different conditions. The colloidal suspension of the nanocomposite was kept: 1) 4 °C for three months; and 3) in DMEM media at a concentration of 0.5 OD/ml, maintained at 37°C for 72 h. Their optical spectra were monitored using a UV-vis spectrophotometer.

2. Result

2.1 Polymer Screening

The optical absorbance of Cit-GNRs (0.32, 0.65, 1.3, 2.6, 5.3, 10.5, and 21 OD/ml) were analyzed using a Nanodrop in the BSA module. A linear curve was generated by plotting the concentration against the absorbance, yielding the linear equation $y = 0.1676x - 0.0855$ ($r = 0.9999$). The quantification of Cit-GNRs absorbance was performed through an external calibration method, enabling the calculation of the Cit-GNRs absorbance. Similarly, the optical absorbance of GNCs (0.5, 1, 2, 5, 8, and 10 mg/ml, based on BSA concentration) was also analyzed using the Nanodrop in the BSA module. A linear curve was plotted by correlating the concentration with the absorbance, resulting in the equation $y = 1.5459x - 0.1176$ ($r = 0.9996$).

Chitosan, PLL and PAH (0.5mg/ml) were individually incubated with Cit-GNRs coated with GNCs (3 OD/ml) for 2 h. This incubation process resulted in the conversion of Cit-GNRs into a positively charged state. Following overnight incubation in a solution containing 5 mg/ml of GNCs and subsequent centrifugation, the quantity of GNCs present on the surface of GNRs was measured and presented in the table below.

Table S1. The quantity of GNCs assembled onto GNRs (mg. GNCs/OD. GNRs).

Polymer	First Layer	Second Layer
Chitosan	0.06 ± 0.03	0.23 ± 0.03
PLL	0.06 ± 0.03	0.21 ± 0.02
PAH	0.06 ± 0.03	0.70 ± 0.14

The quantity of GNCs plays a crucial role in determining the subsequent Doxo-loading content, which relies on the binding sites of BSA conjugated to GNRs. Among PLL, chitosan, and PAH, it was observed that PAH exhibited a greater ability to integrate a higher quantity of GNCs onto GNRs compared to PLL and chitosan. The SPR shift was monitored through UV-Vis-NIR measurements after each deposition step. Following the deposition of chitosan and PLL onto Cit-GNRs, the spectral shift towards the red was less than 3 nm, primarily caused by the deposition of the second layer of GNCs. However, in the case of PAH, the spectral shift exceeded 5 nm. Based on these findings, PAH was selected for further experimentation.

2.2 Cross-link of GNRs-GNCs

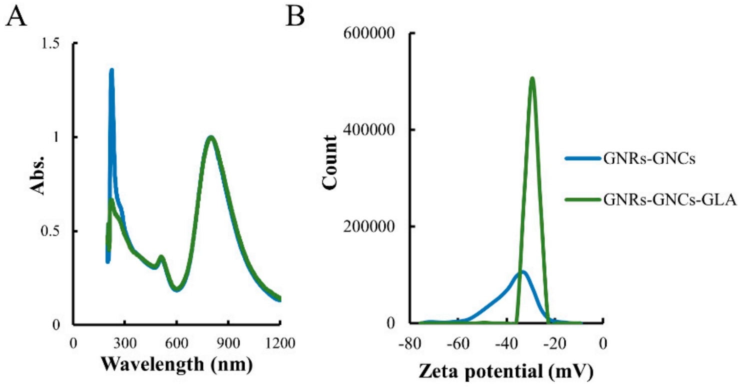

Figure S1. Optical spectra and zeta potential of GNRs-GNCs before and after GLA cross-link. (**A**). The spectra of GNRs with one layer of GNCs before and after GLA cross-linking. (**B**). Zeta potential of GNRs with one layer of GNCs before and after GLA cross-linking.

2.3 Photothermal effect of Cit-GNRs

As presented in Figure S2A,B, the temperature increase was dependent on the Cit-GNRs concentration and the irradiation duration. Higher concentrations and longer irradiation resulted in greater heat generation and higher temperatures. For effective hyperthermia treatment, a local temperature of over 40 °C is required. In this experiment, Cit-GNRs at concentrations of 0.25 and 0.5 OD/ml achieved a temperature of 40 °C within 3-4 min, while 1 OD/ml Cit-GNRs only required 1min. By the end of the 5min irradiation, the solution temperatures for the four concentrations, from low to high, were 35 °C, 42 °C, 47 °C, and 55 °C, respectively. Based on these findings, it was determined that the concentrations of Cit-GNRs at 0.25, 0.5, and 1 OD/ml were appropriate for further experiment in this study, and that the photothermal parameters for MDA-MB-231 cells were set to 400 mW/cm^2 for 4 min.

As Figure S2C presents, Cit-GNRs at concentrations of 0.25 and 0.5 OD/ml had minor effect on the cells, with cellular viability remaining above 90% within a 72 h period. However, in the group treated with 1 OD/ml, there was a significant drop in cellular viability to 50% after 24 h. Following a 72 h incubation period, the cell viability further decreased to only 15%. Importantly, the photothermal effect did not have any impact on cells without nanocomposite treatment, and their cellular viability was comparable to that of control group.

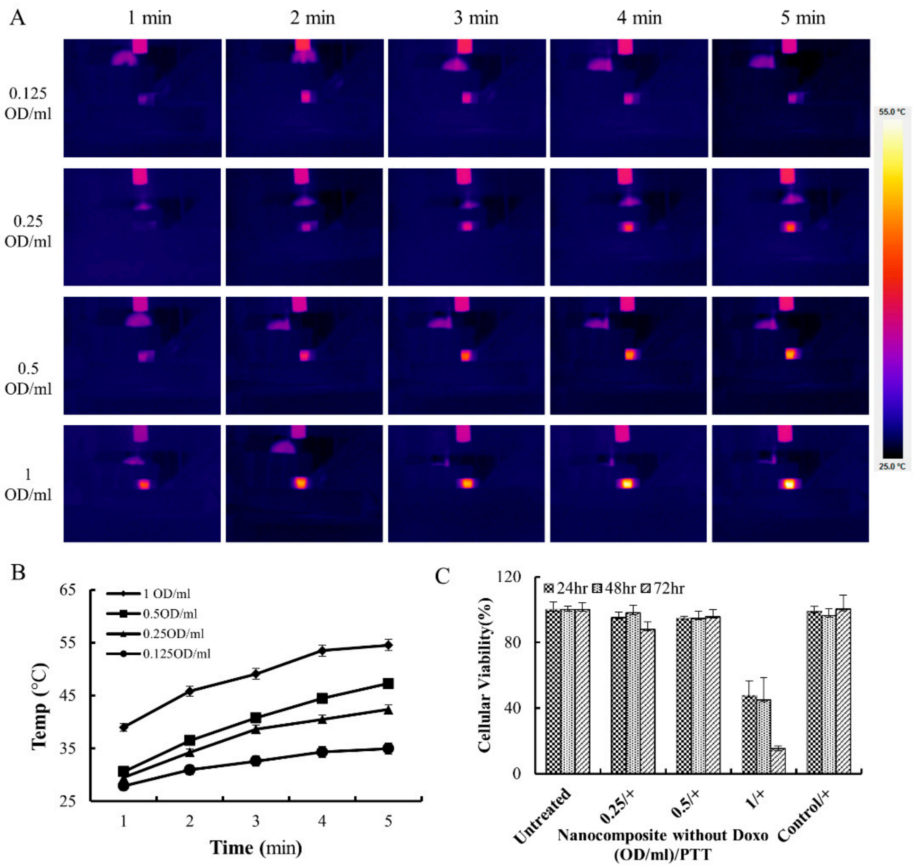

Figure S2. Thermographic images and temperature change of Cit-GNR over time under laser illumination (808 nm, 400 mW/cm^2). (**A**). Thermographic images of Cit-GNRs (0.125, 0.25, 0.5 and 1 OD/ml) over 5 min. The temperature was virtually presented by a color scale, where blue color indicated lower temperatures and bright yellow color indicated higher temperatures, as depicted in the continuous color map on the right. (**B**). Thermographic images and temperature curves of Cit-GNRs (0.125, 0.25, 0.5 and 1 OD/ml) over 5min. (**C**). Thermal effect of Cit-GNRs (0, 0.25, 0.5 and 1 OD/ml) on MDA-MB-231 cells. "+" refers to application of PPT (400 mW/cm^2, 4 min).

2.4 Stability of the nanocomposite

Aggregation of the nanocomposite can lead to the loss of optical properties. To investigate the stability, the nanocomposite was subjected to different conditions and monitored using a UV-Vis-NIR spectrophotometer. After storing the nanocomposite at 4 °C for 3 months, the optical spectrum remained unchanged compared to the initial nanocomposite (Figure S3A), indicating stability for at least 3 months at 4 °C. Similarly, the stability was assessed in DMEM media at 37 °C for 72 h. In the group without photothermal therapy (PPT), the plasmon resonance peaks shifted to the red side and exhibited a slight broadening, most likely due to the formation of a protein corona on the nanocomposite. However, the overall distribution of the plasmon resonance band suggested the stability of the nanocomposite (Figure S3B). In the PPT group, a similar trend was observed with a shift in the plasmon resonance peak, while the general distribution resembled that of the nanocomposite suspended in water (Figure S3C). Over time, the height of the plasmon resonance peaks decreased due to the diminishing concentration of colloids in the medium. The total scattering plasmon resonance peaks might have been obscured by serum components, resulting in reversed peaks as a result of serum consumption by cells.

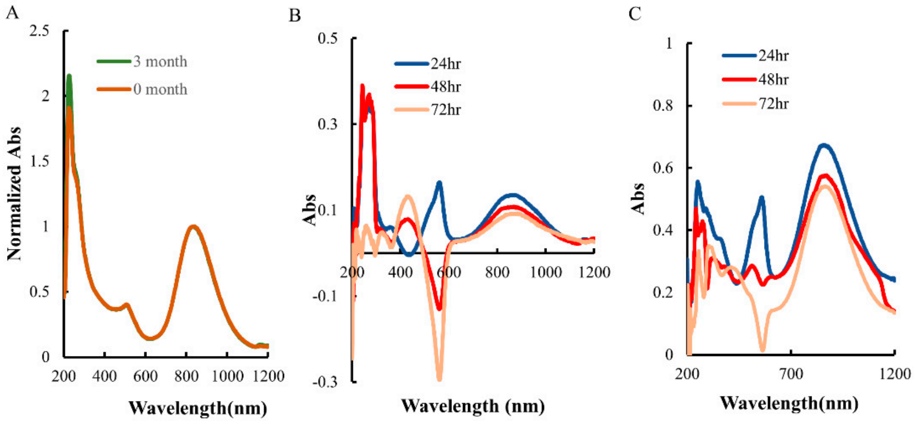

Figure S3. The optical spectra of nanocomposite storage at 4 °C for 3 months. (**A**), 37°C with PPT (**B**), and 37 °C with/o PPT (**C**).
